# Effects of Mechanical Stretching on the Morphology and Cytoskeleton of Vaginal Fibroblasts from Women with Pelvic Organ Prolapse

**DOI:** 10.3390/ijms16059406

**Published:** 2015-04-27

**Authors:** Sumei Wang, Zhenyu Zhang, Dongyuan Lü, Qiuxiang Xu

**Affiliations:** 1 Department of Obstetrics and Gynecology, Beijing Chaoyang Hospital, Capital Medical University, Beijing 100020, China; E-Mails: sumeiwang@ccmu.edu.cn (S.W.); xuqiuxiang2006@163.com (Q.X.); 2 Center of Biomechanics and Bioengineering and Key Laboratory of Microgravity (National Microgravity Laboratory), Institute of Mechanics, Chinese Academy of Sciences, Beijing 100190, China

**Keywords:** cyclical mechanical stretch, estrogen, pelvic organ prolapse, vaginal fibroblasts

## Abstract

Mechanical load and postmenopausal hypoestrogen are risk factors for pelvic organ prolapse (POP). In this study, we applied a 0.1-Hz uniaxial cyclic mechanical stretching (CS) with 10% elongation and 10^−8^ M 17-β-estradiol to vaginal fibroblasts isolated from postmenopausal women with or without POP to investigate the effects of CS and estrogen on cell morphology and cytoskeletons of normal and POP fibroblasts. Under static culture condition, POP fibroblasts exhibited lower cell circularity and higher relative fluorescence intensities (RFIs) of F-actin, α-tubulin and vimentin. When cultured with CS, all fibroblasts grew perpendicular to the force and exhibited a decreased cell projection area, cell circularity and increased cell length/width ratio; normal fibroblasts exhibited increased RFIs of all three types of cytoskeleton, and POP fibroblasts exhibited a decreased RFI of F-actin and no significant differences of α-tubulin and vimentin. After being cultured with 17-β-estradiol and CS, normal fibroblasts no longer exhibited significant changes in the cell projection area and the RFIs of F-actin and α-tubulin; POP fibroblasts exhibited no significant changes in cell circularity, length/width ratio and F-actin even with the increased RFIs of α-tubulin and vimentin. These findings suggest that POP fibroblasts have greater sensitivity to and lower tolerance for mechanical stretching, and estrogen can improve the prognosis.

## 1. Introduction

Pelvic organ prolapse (POP) is a common disorder predominately diagnosed in postmenopausal women [1], with more than 40% of women aged 50–79 years exhibiting a certain degree of POP [2] and approximately 30% of surgical treatment cases requiring reoperation [3], adversely affecting the sufferer’s quality of life. Although many factors, such as vaginal labor, postmenopausal hypo-estrogenic state, advancing age, chronic obstructive lung diseases, long-term constipation, heavy lifting, and genetic predisposition, have been identified as risk factors for POP [4,5,6], the exact etiology and pathogenesis underlying POP remain poorly understood. The female pelvic floor is a special structure that maintains pelvic organs within the body while allowing the passages of the urinary tract, genital tract and rectal hiatus; at the same time, the female pelvic floor is subjected to a constantly changing mechanical load from intra-abdominal pressure and gravity due to woman’s upright activities. The superimposition of these two effects forms the physiological basis of POP. Anatomically, the vagina and its supportive connective tissue provide one of the primary support mechanisms for maintaining the positions of pelvic organs adjacent to the vagina [7]. The connective tissue is a passive viscoelastic “material” that is capable of transferring forces, which allows the tissue to relax and achieve a lower level of resting tension [8,9]. When an external tensile load is applied to the tissue, the fibroblasts can sense the mechanical properties of their environment and actively respond to mechanical stimuli through cytoskeletal remodeling [10,11]; the cytoskeletons not only serve in the maintenance of cellular structure and shape but also can transmit mechanical signals and participate in many cellular functions, including proliferation, apoptosis, and migration [12,13]. We speculate that the healthy and POP fibroblasts in the vaginal wall and pelvic floor connective tissues will present different behaviors when cyclic mechanical stretching (CS) acts on them. Accompanied by the changes in fibroblast morphology, cytoskeletal structure and cellular function, the tissues would constantly remodel and elongate, and eventually result in the occurrence and development of POP. Current information about the effects of mechanical stretching on the cell morphology and cytoskeletons of vaginal fibroblasts and the relationship between these changes and POP is limited. We hypothesized that the stretching forces cause different behaviors in the healthy and POP fibroblasts, as does the postmenopausal decrease in estrogen level. Mechanical loading on fibroblasts* in vitro* is often introduced using a substrate stretching method to mimic the environment* in vivo*, and uniaxial stretching studies have provided much information about the effects of mechanical loads on fibroblasts cultured on a two-dimensional substrate; furthermore, 10% mechanical stretching of fibroblasts is well within the physiologically relevant levels of force normally experienced by tendon fibroblasts* in vivo* [13], and a 0.1-Hz stretching frequency seems likely to mimic the change of intra-abdominal pressure while a woman holds her breath to exert pressure under conditions of labor, constipation, or heavy lifting. Therefore, we applied a 0.1-Hz uniaxial CS force with 10% elongation and administered 10^−8^ M 17-β-estradiol (E_2_) to vaginal fibroblasts derived from postmenopausal women with or without POP. In addition, we selected a 12-h stretching duration every day to mimic a woman’s daily activities in the stretching experiment. This approach was designed to investigate the changes in the cell morphology and the relative fluorescence intensities (RFIs) of the cytoskeletal proteins F-actin, α-tubulin and vimentin using confocal laser scanning microscopy to determine the effects of mechanical stretching on POP fibroblasts and to evaluate the efficacy of estrogen therapy (ET).

## 2. Results

### 2.1. Cell Culture and Identification

**Figure 1:**
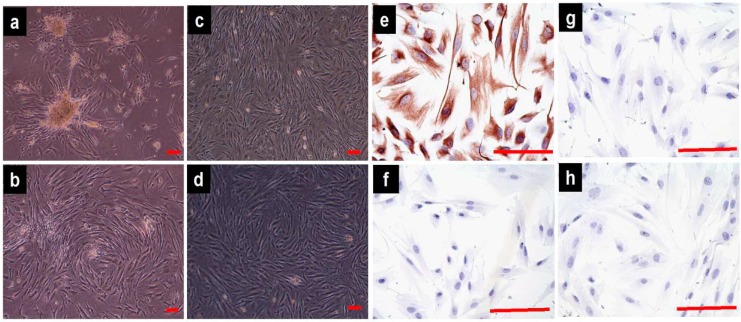
Morphological characteristics and immunocytochemical identification of vaginal fibroblasts (images acquired with an inverted microscope). Primary cultured pelvic organ prolapse (POP) fibroblasts (passage 0) after 72 h (**a**) and 7 days (**b**); POP fibroblasts at passages 4 (**c**) and 5 (**d**). Streptavidin-peroxidase (SP) immunohistochemical staining of POP fibroblasts (passage 4): The characteristic staining of vimentin (**e**), cytokeratin (**f**), α-smooth muscle actin (**g**), and negative control (**h**). Bar = 100 μm.

The isolated fibroblasts from the vaginal wall connective tissues exhibited stellate, bipolar, and spindle-shaped characteristics under an inverted microscope (Figure 1a–d); no differences were observed between the POP and control groups. The streptavidin-peroxidase (SP) immunohistochemical staining of isolated cells from the two groups revealed strong cytoplasmic expression of vimentin, with an index of staining (IS) value of vimentin being 12 (+++); the IS value of cytokeratin was 0 (−); the IS value of α-smooth muscle actin was 0 to 1 (−); and the IS value of the negative control was 0 (−). These outcomes confirmed the origin of the connective tissue fibroblasts (Figure 1e–h).

### 2.2. Effects of Cyclic Mechanical Stretching and 17-β-Estradiol (E_2_) on Vaginal Fibroblast Proliferation

After the vaginal fibroblasts were seeded at a density of 2 × 10^4^/cm^2^ and exposed to the experimental conditions for 72-h periods, the viable cells in each group were collected and counted. There was a total of 6 groups, including the control group (C) and the pelvic organ prolapse group (P) without CS and E_2_ [(C-E_2_-CS) and (P-E_2_-CS)], groups C and P without E_2_ but with CS [(C-E_2_+CS) and (P-E_2_+CS)], groups C and P with E_2_ but without CS [(C+E_2_-CS) and (P+E_2_-CS)], and groups C and P with CS and E2 [(C+E_2_+CS) and (P+E_2_+CS)]. No significant difference was observed between (P-E_2_-CS) and (C-E_2_-CS) ((2.69 ± 0.29) × 10^5^* vs.* (2.07 ± 0.29) × 10^5^, *p* = 0.16). Stretching did not produce a significant effect on the cell proliferation of normal or POP fibroblasts; no significant difference was observed between (C-E_2_+CS) and (C-E_2_-CS) ((2.24 ± 0.33) × 10^5^* vs.* (2.07 ± 0.29) × 10^5^, *p* = 0.70) or between (P-E_2_+CS) and (P-E_2_-CS) ((2.25 ± 0.31) × 10^5^* vs.* (2.69 ± 0.29) × 10^5^, *p* = 0.32). The administration of E_2_ in the presence of stretching resulted in similar findings; there was no significant difference between (C+E_2_+CS) and (C+E_2_-CS) ((1.94 ± 0.25) × 10^5^* vs.* (2.28 ± 0.36) × 10^5^, *p* = 0.48) or between (P+E_2_+CS) and (P+E_2_-CS) ((2.43 ± 0.37) × 10^5^* vs.* (2.29 ± 0.32) × 10^5^, *p* = 0.75).

### 2.3. Effects of Cyclic Mechanical Stretching and E_2_ on Vaginal Fibroblast Morphology

**Figure 2:**
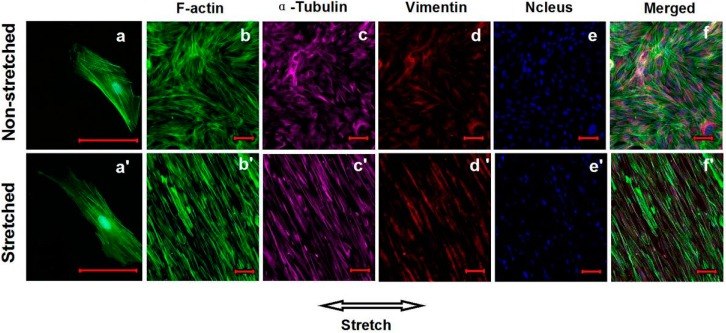
Effects of cyclic mechanical stretching (CS) on the orientations of cytoskeletons and vaginal fibroblasts. The static growth F-actin stress fibers (**a**) and fibroblasts (**b**–**f**); The stretching growth F-actin stress fibers (**a**') and fibroblasts (**b**'–**f**'). Bar = 100 μm.

At the end of the 72-h experimental periods, a morphological analysis of the stretching cultured fibroblasts revealed that their F-actin stress fibers had assumed a crescent morphology and aligned with the long axis of the cell bodies (Figure 2a'), and the stretching cultured fibroblasts aligned perpendicular to the force and exhibited a wider intercellular space (Figure 2b'–f'). Under the static culture condition, the stress fibers of the fibroblasts were straight and randomly oriented (Figure 2a), and the fibroblasts exhibited a random and tight distribution independent of the surface (Figure 2b–f).

**Figure 3:**
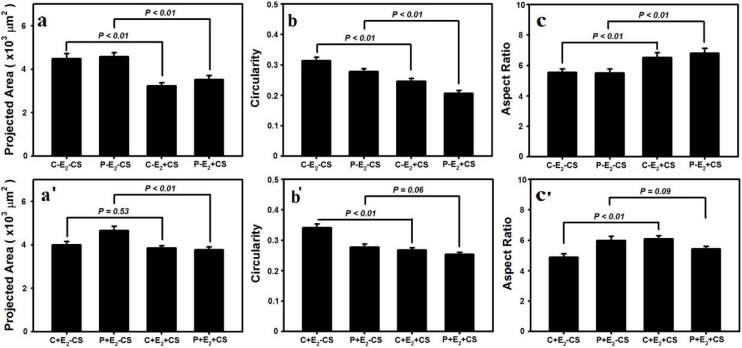
Effects of CS and 17-β-estradiol (E_2_) on the vaginal fibroblast projection area, circularity and length/width ratio (**a**–**c**, **a**'–**c**'). Data are represented as the mean ± SE from triplicate trials.

Through an analysis of single-cell images photographed by confocal laser scanning microscopy using Image J software, we observed that the cell circularity was significantly lower in (P-E_2_-CS) compared with (C-E_2_-CS) (0.278 ± 0.009* vs.* 0.314 ± 0.011, *p* < 0.01); there was no significant difference in the cell projection area (μm^2^) or the cell length/width ratio between the two groups (4579 ± 183* vs.* 4481 ± 234, *p* = 0.69; 5.51 ± 0.25* vs.* 5.54 ± 0.22, *p* = 0.93). Following the application of stretching, both the normal and POP fibroblasts became smaller, irregular, and longer. Our data indicated that the cell projection area (μm^2^) was significantly lower in (C-E_2_+CS) compared with (C-E_2_-CS) (3232 ± 139* vs.* 4481 ± 234, *p* < 0.01) as well as in (P-E_2_+CS) compared with (P-E_2_-CS) (3520 ± 181* vs.* 4579 ± 183, *p* < 0.01) (Figure 3a). Furthermore, the cell circularity was significantly lower in (C-E_2_+CS) compared with (C-E_2_-CS) (0.246 ± 0.009* vs.* 0.314 ± 0.011, *p* < 0.01) as well as in (P-E_2_+CS) compared with (P-E_2_-CS) (0.207 ± 0.009* vs.* 0.278 ± 0.009, *p* < 0.01) (Figure 3b). With respect to the cell length/width ratio, there was a significant increase in (C-E_2_+CS) compared with (C-E_2_-CS) (6.52 ± 0.32* vs.* 5.54 ± 0.22, *p* < 0.01) as well as in (P-E_2_+CS) compared with (P-E_2_-CS) (6.79 ± 0.32* vs.* 5.51 ± 0.25, *p* < 0.01) (Figure 3c). In cells treated with E_2_ in the presence of stretching, normal fibroblasts no longer exhibited a significant change in the cell projection area (μm^2^), and no significant difference was observed between (C+E_2_+CS) and (C+E_2_-CS) (3846 ± 117* vs.* 3992 ± 164, *p =* 0.53); however, the cell projection area (μm^2^) of POP fibroblasts still became smaller, and a significant difference was observed between (P+E_2_+CS) and (P+E_2_-CS) (3772 ± 129* vs.* 4657 ± 189, *p* < 0.01) (Figure 3a'). With respect to cell circularity, although there was a significant decrease in (C+E_2_+CS) compared with (C+E_2_-CS) (0.267 ± 0.008* vs.* 0.341 ± 0.013, *p* < 0.01), no significant difference was observed between (P+E_2_+CS) and (P+E_2_-CS) (0.253 ± 0.007* vs.* 0.277 ± 0.010, *p* = 0.06) (Figure 3b'). With respect to the cell length/width ratio, there was a significant increase in (C+E_2_+CS) compared with (C+E_2_-CS) (6.08 ± 0.21* vs.* 4.88 ± 0.23, *p* < 0.01), but there was no longer a significant difference between (P+E_2_+CS) and (P+E_2_-CS) (5.42 ± 0.17* vs.* 5.97 ± 0.28, *p* = 0.09) (Figure 3c').

### 2.4. Effects of Cyclic Mechanical Stretching and E_2_ on Vaginal Fibroblast Cytoskeletons

**Figure 4:**
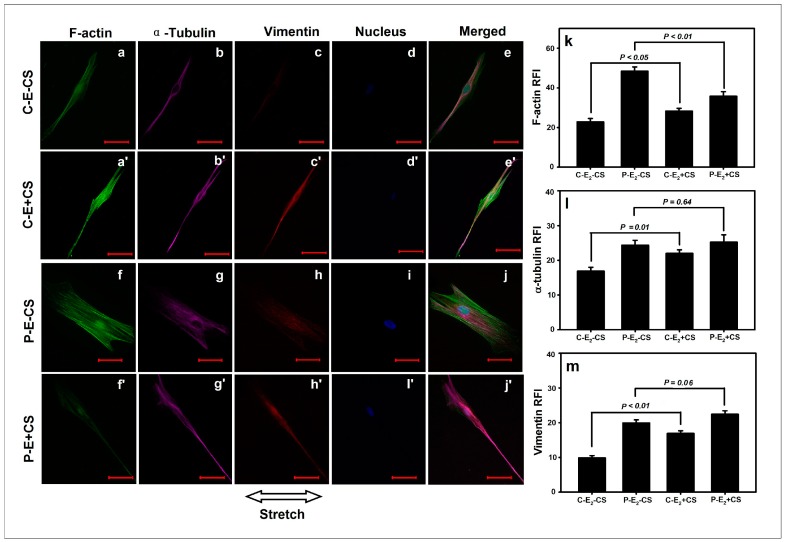
Effects of CS on the cytoskeletons of vaginal fibroblasts. The comparisons in relative fluorescence intensities (RFIs) of the cytoskeletal proteins between the static-cultured fibroblasts and the CS-cultured fibroblasts (**k**–**m**). Images of the cytoskeletons (**a'**–**e'** and **a**–**e**, **f'**–**j'** and **f**–**j**). Bar = 50 μm. The RFIs of F-actin (**k**), α-tubulin (**l**), and vimentin (**m**) were measured and are represented as the mean ± SE from triplicate trials.

The expression of the cytoskeletal protein F-actin, α-tubulin, and vimentin was semiquantified using RFI. Compared with (C-E_2_-CS), the RFI of F-actin in (P-E_2_-CS) was significantly elevated (48.3 ± 2.2* vs.* 22.8 ± 1.7, *p* < 0.01), which was similar to the differences in α-tubulin and vimentin between the two groups (24.3 ± 1.4* vs.* 16.9 ± 1.1, *p* < 0.01; 19.9 ± 0.9* vs.* 9.9 ± 0.6, *p* < 0.01). Following the application of stretching, normal fibroblasts exhibited significant increases in the expressions of all three cytoskeletons; in addition to the data demonstrate a significant difference in the RFI of F-actin between (C-E_2_+CS) and (C-E_2_-CS) (28.2 ± 1.5* vs.* 22.8 ± 1.68, *p* < 0.05), significant differences in α-tubulin and vimentin between these two groups (22.0 ± 1.0* vs.* 16.9 ± 1.1, *p* = 0.01; 16.9 ± 0.7* vs.* 9.9 ± 0.6, *p* < 0.01) were also observed (Figure 4a'–e', a–e, k–m). However, the POP fibroblasts subjected to stretching forces exhibited a significant decrease in the expression of F-actin; the data revealed a significant difference in the RFI of F-actin between (P-E_2_+CS) and (P-E_2_-CS) (35.8 ± 2.3* vs.* 48.3 ± 2.2, *p* < 0.01), and no significant differences were observed in the RFIs of α-tubulin and vimentin between these two groups (25.2 ± 2.1* vs.* 24.3 ± 1.4, *p* = 0.64; 22.4 ± 1.0* vs.* 19.9 ± 0.9, *p* = 0.06) (Figure 4f'– j', f–j, k–m).

**Figure 5:**
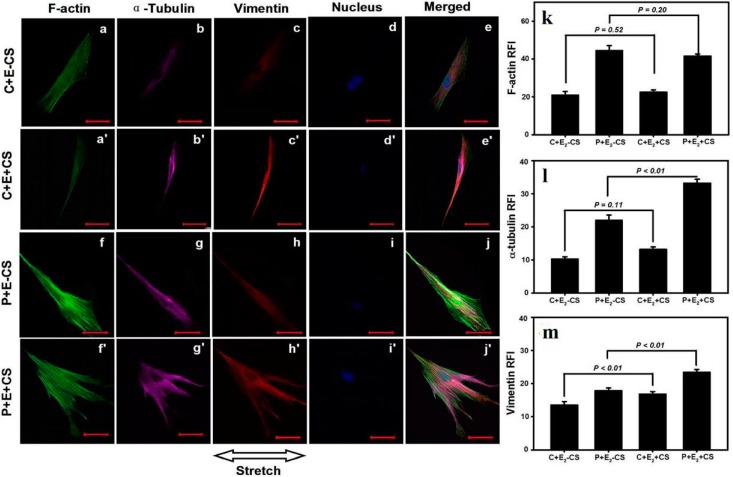
Effects of CS and E_2_ on vaginal fibroblast cytoskeletons. The comparisons in RFIs of the cytoskeletal proteins between the fibroblasts cultured in static conditions with E_2_ and cultured in the presence of CS concomitant E_2_ (**k**–**m**). Images of the cytoskeletons (**a'**–**e'** and **a**–**e**, **f'**–**j'** and **f**–**j**). Bar = 50 μm. The RFIs of F-actin (**k**), α-tubulin (**l**), and vimentin (**m**) were measured and are represented as the mean ± SE from triplicate trials.

When cultured in the presence of E_2_, the expressions of F-actin and α-tubulin in normal fibroblasts no longer exhibited significant changes in response to stretching, with the data exhibiting no significant differences in the RFIs between (C+E_2_+CS) and (C+E_2_-CS) (22.5 ± 1.2* vs.* 20.9 ± 2.0, *p* = 0.52; 13.2 ± 0.8* vs.* 10.3 ± 0.6, *p* = 0.11), although the RFI of vimentin in (C+E_2_+CS) remained significantly increased relative to (C+E_2_-CS) (16.8 ± 0.8* vs.* 13.5 ± 1.1, *p* < 0.01) (Figure 5a'–e' and a–e, k–m). The application of E_2_ to POP fibroblasts abrogated the significant decrease in the expression of F-actin induced by CS; the data indicated a non-significant difference between (P+E_2_+CS) and (P+E_2_-CS) (41.5 ± 1.2* vs.* 44.5 ± 2.6, *p* = 0.20). Both α-tubulin and vimentin exhibited significantly higher expressions in POP fibroblasts in the presence of E_2_ and CS; the RFIs differed significantly between (P+E_2_+CS) and (P+E_2_-CS) (33.2 ± 1.2* vs.* 22.0 ± 1.6, *p* < 0.01; 23.4 ± 0.9* vs.* 17.9 ± 0.8, *p* < 0.01) (Figure 5f'–j', f–j, k–m).

## 3. Discussion

In this study, our data revealed that the vaginal fibroblasts grew perpendicular to the orientation of the stretching force under conditions of CS and their F-actin stress fibers aligned to the cells’ long axis. These results are consistent with previous studies [11,14]. It has been established that the mechanical stretching load applied to connective tissues can induce conformational changes in the extracellular matrix (ECM), activate integrins, achieve mechanical signal transduction from the ECM to the cytoskeleton and then the nucleus, and eventually cause gene transcription, protein translation and modulation of the tissue behavior [12,13,15]. The ability of fibroblasts to acquire stress fibers and differentiate into myofibroblasts to drive tissue repair by secreting collagen and reorganizing the ECM was confirmed during wound healing [16].

As viscoplastic materials [17,18], the vaginal connective tissue should have viscoplastic properties with mechanical loading and unloading curves showing stress relaxation, creep and hysteresis; the mechanical stretching of connective tissues should be accompanied by the remodeling of cellular morphology. Our data indicate that the stretching cultured fibroblasts became smaller, irregular, and longer. These changes, together with the non-significant change in cell proliferation, resulted in wider intercellular spaces. When this series of fibroblast remodeling in one direction and the accompanied changes in cell functions appearing in the vaginal and other pelvic floor connective tissues continues for a long time, the tissues may constantly grow in an elongated way and eventually present laxity, and then POP may occur. Compared with fibroblasts growing in a random and tight distribution under static culture conditions, the stretched growing connective tissues may exhibit relatively lower strength. Our study also investigated the effects of CS on the semiquantitative expressions of the three main types of cytoskeletal proteins, including F-actin microfilaments, α-tubulin microtubules and vimentin intermediate filaments, in the cultured vaginal connective tissue fibroblasts. Nearly all cells contain an interconnected cytoskeletal system within their viscous cytosol; the tensional forces are borne by the microfilaments and intermediate filaments and balanced by the internal microtubule struts and external ECM adhesions [19]. In addition to playing a role in mechanical signal transduction, microfilaments are responsible for the cell shape, microtubules are responsible for intracellular transport and the formation of mitotic spindles, and intermediate filaments are mainly associated with supporting and anchoring the position of the nucleus and organelles in the cytosol [20]. Our results found that static-cultured POP fibroblasts exhibited significantly higher expression of F-actin, α-tubulin, or vimentin, which suggested that POP fibroblasts possess higher mechanosensitivity and revealed active responses from cytoskeletons to the tension existing on the culture substrate. When a frequency of 0.1 Hz and an elongation of 10% uniaxial CS was applied in our study, normal fibroblasts exhibited higher expressions of the three cytoskeletal components, whereas POP fibroblasts exhibited a significant decrease in the expression of F-actin and non-significant changes in the expression of α-tubulin and vimentin. These findings may suggest that POP fibroblasts have a lower tolerance for stretching forces, as their mechanical stretching properties have already reached their limits due to the long-term and excessive stretching load caused by pregnancy, delivery, and intra-abdominal pressure. Under this condition, overloading stretching force will destroy the cytoskeletal system and affect the shape and metabolic function of POP fibroblasts. Ewies* et al.* [21] have identified many morphological abnormalities of the cytoskeleton and cell shape during overloading stretching. In this study, full-thickness vaginal wall samples were procured from the anterior wall near the vaginal apex to shield from the confounding secondary effects of prolapse [22] and to represent the supportive connective tissues of the pelvic floor which provide part of the anatomic support for the cervix and the upper vagina [7].

It is likely that the stretching conditions, together with the postmenopausal hypo-estrogenic state, eventually affect the mechanical properties of the pelvic supportive connective tissues and result in POP. Clinically, the long-term and excessive increase of intra-abdominal pressure can be directly transmitted to the vaginal wall to increase the mechanical load and cause tissue stretching; the overdistension of the vagina associated with vaginal delivery and atrophic changes associated with aging and menopause were considered to result in a decrease in vaginal tissue resilience and POP [23,24,25,26,27]. Estrogen therapy (ET) has long been used to improve the symptoms of POP; however, the precise effect of estrogen on the pelvic floor structure and its role in the prevention and treatment of POP remain controversial. Liu* et al.* [28] deemed that ET was an ineffective POP treatment, and Ewies* et al.* [21] reported that the use of E_2_ increased fibroblast proliferation. The existence of estrogen receptors in the pelvic tissues of postmenopausal women [29,30], have led us to believe the effects of ET. Our data indicated that CS and E_2_ did not produce significant effect on the proliferation of POP or normal fibroblasts, but affected the cytoskeletons and cell morphology. We found that when E_2_ concomitant CS was applied in this study, normal fibroblasts no longer exhibited significant changes in the cell projection area and the expressions of F-actin and α-tubulin, and POP fibroblasts no longer exhibited significant changes in the cell circularity, length/width ratio and the expression of F-actin, but exhibited significant increases in the expression of α-tubulin and vimentin. Here, E_2_ inhibited the over-expressions of F-actin and α-tubulin in healthy fibroblasts and the decreased expression of F-actin and α-tubulin in POP fibroblasts induced by a mechanical stretching load to restrain cell deformation; the action of E_2_ maintaining the high expression of vimentin is important for supporting and anchoring the position of the nucleus and organelles in the cytosol. These results suggest that estrogen’s regulation of the effect of mechanical stretching on pelvic floor fibroblasts is likely to be dual directional. ET is beneficial for maintaining the integrity and functions of fibroblasts and the connective tissues during mechanical stretching to prevent and improve the prognosis of POP.

## 4. Experimental Section

### 4.1. Patient Selection and Tissue Collection

This study was approved by the medical ethics committee of Beijing Chaoyang Hospital, Capital Medical University on 23 January 2013 (The project identification code: 2013-1-23). We recruited a total of ten participants: Five women (aged 53–69 years) with advanced POP (stage III–IV by POP quantification) [31] were included in the POP group, and five women (aged 52–67 years) without POP were included as controls. All participants provided verbal and written informed consent. Women with a history of endometriosis, gynecologic malignancies, pelvic inflammatory conditions, connective tissue disorders, or emphysema were excluded. After informed consent was obtained, a 1-cm^2^ area of full-thickness vaginal wall was procured from the anterior wall near the vaginal apex during pelvic floor construction surgery for advanced POP. Full-thickness vaginal wall samples of the same size were also obtained from the same anatomic position in control women during their benign gynecological hysterectomy for fibroids, dysfunctional bleeding, or ovarian cysts.

### 4.2. Primary Culture of Human Vaginal Fibroblasts

Human vaginal fibroblasts were isolated from the fresh vaginal wall samples. After excision from the donor, the samples were immediately placed in 4 °C sterile Dulbecco’s phosphate-buffered saline (DPBS, Hyclone, South Logan, UT, USA) with 1% penicillin/streptomycin (P/S, Hyclone, South Logan, UT, USA) and were sent to the laboratory within 2 h. The connective tissue blocks from the vaginal wall samples were separated and minced into 1-mm^3^ cubes, digested with 0.5% collagenase type I (Sigma-Aldrich, St. Louis, MO, USA) in Dulbecco’s modified Eagle’s medium (DMEM, Gibco, Grand Island, NY, USA) with gentle rotation and placed in a 5% CO_2_ humidified incubator at 37 °C overnight. The fine sand-like tissue pieces formed by collagenase digestion were suspended in DMEM supplemented with 10% fetal bovine serum (FBS, Gibco, Grand Island, NY, USA) and 1% P/S, and then centrifuged at 1500 rpm. The supernatant was removed, and the sediments were reconstituted with 5 mL DMEM (supplemented with 10% FBS and 1% P/S) and transferred to a 25-cm^2^ polystyrene petri dish (Corning Coster Co., Cambridge, MA, USA) for culture until the vaginal fibroblasts reached 90% confluence, at which time the fibroblasts were sub-cultured. After identifying the vaginal fibroblasts at their fourth passage, the cells were collected by trypsin digestion and used in the following experiments.

### 4.3. Phenotype Identification of the Vaginal Fibroblasts

The derived cells were confirmed to be of vaginal connective tissue fibroblastic origin using the SP immunohistochemical method. Cells were cultured in chamber slides to 50% confluence and were subsequently washed, fixed, and treated with 0.4% Triton X-100. Endogenous peroxidase activity was blocked with 3% hydrogen peroxide. The cells were incubated with specific primary antibodies, including mouse anti-human vimentin monoclonal antibody, mouse anti-human cytokeratin (spectrum) monoclonal antibody, and mouse anti-human α-smooth muscle actin monoclonal antibody (all from Zhongshan Goldbridge Biotechnology, Beijing, China) at 37 °C overnight; DPBS was used as negative control in place of the primary antibody. The sections were then incubated with the PV-6000 polymer detection system (Zhongshan Goldbridge Biotechnology, Beijing, China) for immunohistological staining, and the immunoreactivity was revealed using a 3,3'-diaminobenzidine tetrahydrochloride substrate kit (Zhongshan Goldbridge Biotechnology, Beijing, China) as the final chromogen. Finally, the sections were counterstained with Meyer’s hematoxylin. According to the suggestions of Xu* et al.* [32] and Zhang* et al.* [33], we used IS to assess the staining results in this study: IS = the percentages of positive cells × standard scores of the staining intensity. The relative populations of the positive cells were assessed under the same objective: Negative, 0 points; ≤10%, 1 point; 11%–50%, 2 points; 51%–75%, 3 points; and >75%, 4 points. The standard scores of staining intensity were as follows: No staining, 0 points; bright yellow, 1 point; brown-yellow, 2 points; and brown, 3 points. IS values ≤1 were identified as (−), 2–3 as (+), 4 as (++), and ≥5 as (+++); results between + and +++ indicate the presence of expression.

### 4.4. Loading of Cyclic Mechanical Stretch and the Administration of E_2_

**Figure 6:**
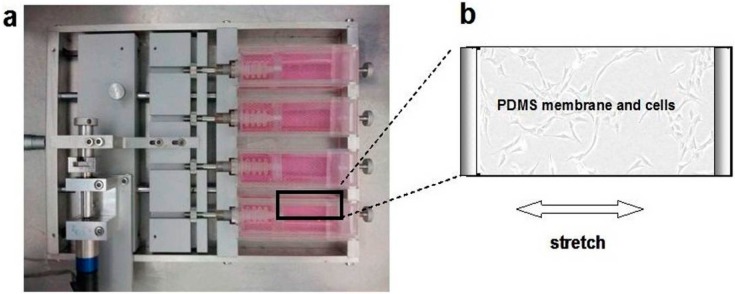
The uniaxial CS was applied to the vaginal fibroblasts via the PDMS membrane fixed in-house of the cell stretching device (**a**); Vaginal fibroblasts sub-cultured on the PDMS membrane (**b**). Arrow indicates the stretching direction.

A 0.1-Hz uniaxial CS with 10% elongation and a 12-h stretching duration every day was performed. The cell stretching device was designed and manufactured to apply stress* in vitro *[34] in this study and consisted of a holder box, a motor, and an elastic membrane with a utilized area of 40 × 20 mm^2^ (length × width) and a thickness of 3 mm. This cell stretching device was designed to apply 0%–30% strain in a uniaxial or equiaxial stretching manner to the cells seeded on the membrane and to take images of the live cells using an inverted microscope (Figure 6a,b), the membrane was made of poly-dimethylsiloxane (PDMS) gel (Sylgard 184, Dow Corning, Midland, MI, USA), and ANSYS software (ANSYS Inc., Pittsburgh, PA, USA) was used to simulate the stress distribution on the elastic membrane when strain was applied to the elastic membrane. In this study, the PDMS membranes were treated with oxygenized plasma and coated with 0.1% gelatin (Sigma-Aldrich, St. Louis, MO, USA) at 37 °C for 2 h before the cells were seeded. The selection of gelatin coating was to utilize its characteristic of denatured collagen [35] to avoid interference from the collagen coating in the observation of collagen molecule secreted by fibroblasts. Twenty-four hours before the application of the indicated conditions, the vaginal fibroblasts were seeded at a density of 2 × 10^4^/cm^2^ for a proliferation assay and at a density of 2 × 10^3^/cm^2^ for immunofluorescent microscopy. A dose of 10^−8^ M E_2_ (Sigma-Aldrich Co, St. Louis, MO, USA) was administered to study the effects of estrogen concomitant with CS. The dose of 10^−8^ M E_2_ is within the physiological range [22,28].

### 4.5. Fibroblast Counting

The effect of CS on the proliferation of vaginal fibroblasts was determined by counting the number of cells. After being seeded on PDMS membranes for 24 h and exposed to the experimental conditions for the next 72 h, the fibroblasts were washed twice with 37 °C pre-warmed DPBS, trypsinized, and resuspended in the culture medium. Subsequently, the resultant fibroblast suspensions were transferred to 1.5-mL microcentrifuge tubes, and cells were counted using the Millipore Scepter 2.0 Handheld Automated Cell Counter (Millipore, Billerica, MA, USA), which can automatically recognize and display the number of viable cells.

### 4.6. Immunological Staining and Imaging with Confocal Microscopy

The fibroblasts from each group were triple-fluorescence stained by fluorescently labeled conjugated antibodies as previously described [36]. After being rinsed with DPBS, fixed with 4% fresh paraformaldehyde and treated with 0.4% Triton X-100, the fibroblasts were incubated with FITC-conjugated phalloidin (Enzo Life Sciences, Farmingdale, NY, USA), Alexa Fluor 555-conjugated α-tubulin (Cell Signaling Technology, Danvers, MA, USA), and Alexa Fluor 647-conjugated vimentin rabbit monoclonal antibodies (Cell Signaling Technology , Danvers, MA, USA) at 4 °C overnight. Finally, the fibroblasts were incubated with Hoechst 33342 (Enzo Life Sciences, Farmingdale, NY, USA) and stored at 4 °C for examination by confocal laser scanning microscopy (Zeiss L710, Carl Zeiss, Dresden, Saxony, Germany). F-actin staining was used to identify the contours of the fibroblasts. For the topographical substrate from each experiment, no less than 40 cells were randomly collected and analyzed. The cell projection area, circularity, length/width ratio, and RFIs of F-actin, α-tubulin, and vimentin were measured using Image J software (National Institutes of Health, Bethesda, MA, USA).

### 4.7. Statistical Analysis

All quantitative data are expressed as the mean ± SE. Statistical comparisons of multiple groups were made using one-way analysis of variance (ANOVA), and the differences between two groups were determined using Student’s *t*-test. Differences were considered statistically significant at the 0.05 level (*p* < 0.05). Each experiment was performed in triplicate, and the researcher who collected and processed the original data was blinded to the group allocation to avoid subjective bias in reporting and interpreting the data.

## 5. Conclusions

In conclusion, POP fibroblasts possess a higher sensitivity to mechanical stimuli and a lower tolerance for mechanical stretching. The administration of E_2_ can improve the mechanical properties of the connective tissue by suppressing the excessive and abnormal remodeling of the fibroblasts and their cytoskeletons. These findings provide new insights into understanding the etiology and pathogenesis of POP and provide positive evidence for the use of ET in POP patients.

## References

[1] Mokrzycki M.L., Mittal K., Smilen S.W., Blechman A.N., Porges R.F., Demopolous R.I. (1997). Estrogen and progesterone receptors in the uterosacral ligament. Obstet. Gynecol..

[2] Hendrix S.L., Clark A., Nygaard I., Aragaki A., Barnabei V., McTiernan A. (2002). Pelvic organ prolapse in the Women’s Health Initiative: Gravity and gravidity. Am. J. Obstet. Gynecol..

[3] Olsen A.L., Smith V.J., Bergstrom J.O., Colling J.C., Clark A.L. (1997). Epidemiology of surgically managed pelvic organ prolapse and urinary incontinence. Obstet. Gynecol..

[4] Jelovsek J.E., Maher C., Barber M.D. (2007). Pelvic organ prolapse. Lancet.

[5] Kim C.M., Jeon M.J., Chung D.J., Kim S.K., Kim J.W., Bai S.W. (2007). Risk factors for pelvic organ prolapse. Int. J. Gynaecol. Obstet..

[6] Word R.A., Pathi S., Schaffer J.I. (2009). Pathophysiology of pelvic organ prolapse. Obstet. Gynecol. Clin. N. Am..

[7] DeLancey J.O. (1992). Anatomic aspects of vaginal eversion after hysterectomy. Am. J. Obstet. Gynecol..

[8] Langevin H.M., Bouffard N.A., Fox J.R., Palmer B.M., Wu J., Iatridis J.C., Barnes W.D., Badger G.J., Howe A.K. (2011). Fibroblast cytoskeletal remodeling contributes to connective tissue tension. J. Cell. Physiol..

[9] Provenzano P., Lakes R., Keenan T., Vanderby R. (2001). Nonlinear ligament viscoelasticity. Ann. Biomed. Eng..

[10] Buck R.C. (1980). Reorientation response of cells to repeated stretch and recoil of the substratum. Exp. Cell Res..

[11] Kong D., Ji B., Dai L. (2008). Stability of adhesion clusters and cell reorientation under lateral cyclic tension. Biophys. J..

[12] Chiquet M., Gelman L., Lutz R., Maier S. (2009). From mechanotransduction to extracellular matrix gene expression in fibroblasts. Biochim. Biophys. Acta.

[13] Wang J.H., Thampatty B.P., Lin J.S., Im H.J. (2007). Mechanoregulation of gene expression in fibroblasts. Gene.

[14] Ruiz-Zapata A.M., Kerkhof M.H., Zandieh-Doulabi B., Brölmann H.A., Smit T.H., Helder M.N. (2013). Fibroblasts from women with pelvic organ prolapse show differential mechanoresponses depending on surface substrates. Int. Urogynecol. J..

[15] Lelièvre S.A. (2009). Contributions of extracellular matrix signaling and tissue architecture to nuclear mechanisms and spatial organization of gene expression control. Biochim. Biophys. Acta.

[16] Tomasek J.J., Gabbiani G., Hinz B., Chaponnier C., Brown R.A. (2002). Myofibroblasts and mechano-regulation of connective tissue remodelling. Nat. Rev. Mol. Cell Biol..

[17] Goh J.T. (2003). Biomechanical and biochemical assessments for pelvic organ prolapse. Curr. Opin. Obstet. Gynecol..

[18] Rubod C., Brieu M., Cosson M., Rivaux G., Clay J.C., de Landsheere L., Gabriel B. (2012). Biomechanical properties of human pelvic organs. Urology.

[19] Ingber D.E., Tensegrity I. (2003). Cell structure and hierarchical systems biology. J. Cell Sci..

[20] Alberts B., Johnson A., Lewis J., Raff M., Roberts K., Walter P. (2002). Molecular Biology of the Cell.

[21] Ewies A.A., Elshafie M., Li J., Stanley A., Thompson J., Styles J., White I., Al-Azzawi F. (2008). Changes in transcription profile and cytoskeleton morphology in pelvic ligament fibroblasts in response to stretch: The effects of estradiol and levormeloxifene. Mol. Hum. Reprod..

[22] Zong W., Jallah Z.C., Stein S.E., Abramowitch S.D., Moalli P.A. (2010). Repetitive mechanical stretch increases extracellular collagenase activity in vaginal fibroblasts. Female Pelvic Med. Reconstr. Surg..

[23] Martins P., Lopes Silva-Filho A., Maciel da Fonseca R., Santos A., Santos L., Mascarenhas T., Natal Jorge R.M., Ferreira A.J. (2013). Biomechanical properties of vaginal tissue in women with pelvic organ prolapse. Gynecol. Obstet. Investig..

[24] DeLancey J.O. (1993). Anatomy and biomechanics of genital prolapse. Clin. Obstet. Gynecol..

[25] Al-Taher H., Sutherst J.R., Richmond D.H., Brown M.C. (1987). Vaginal pressure as an index of intra-abdominal pressure during urodynamic evaluation. Br. J. Urol..

[26] Mouritsen L., Hulbaek M., Brostrøm S., Bogstad J. (2007). Vaginal pressure during daily activities before and after vaginal repair. Int. Urogynecol. J..

[27] O’Dell K.K., Morse A.N., Crawford S.L., Howard A. (2007). Vaginal pressure during lifting, floor exercises, jogging, and use of hydraulic exercise machines. Int. Urogynecol. J..

[28] Liu Y.M., Choy K.W., Lui W.T., Pang M.W., Wong Y.F., Yip S.K. (2006). 17 β-estradiol suppresses proliferation of fibroblasts derived from cardinal ligaments in patients with or without pelvic organ prolapse. Hum. Reprod..

[29] Chen G.D., Oliver R.H., Leung B.S., Lin L.Y., Yeh J. (1999). Estrogen receptor α and β expression in the vaginal walls and uterosacral ligaments of premenopausal and postmenopausal women. Fertil. Steril..

[30] Ewies A.A., Thompson J., Al-Azzawi F. (2004). Changes in gonadal steroid receptors in the cardinal ligaments of prolapsed uteri: immunohistomorphometric data. Hum. Reprod..

[31] Bump R.C., Mattiasson A., Bø K., Brubaker L.P., DeLancey J.O., Klarskov P., Shull B.L., Smith A.R. (1996). The standardization of terminology of female pelvic organ prolapse and pelvic floor dysfunction. Am. J. Obstet. Gynecol..

[32] Xu L.Z., Yang W.T. (1996). Judging standard of immunohistochemical results. Chin. Oncol..

[33] Zhang R.G., Wang C.S., Gao C.F. (2012). Prevalence and pathogenesis of Barrett’s esophagus in Luoyang, China. Asian Pac. J. Cancer Prev..

[34] Lü D., Liu X., Gao Y., Huo B., Kang Y., Chen J., Sun S., Chen L., Luo X., Long M. (2013). Asymmetric migration of human keratinocytes under mechanical stretch and cocultured fibroblasts in a wound repair model. PLoS ONE.

[35] Sbardella D., Fasciglione G.F., Gioia M., Ciaccio C., Tundo G.R., Marini S., Coletta M. (2012). Human matrix metalloproteinases: An ubiquitarian class of enzymes involved in several pathological processes. Mol. Asp. Med..

[36] Lü D., Luo C., Zhang C., Li Z., Long M. (2014). Differential regulation of morphology and stemness of mouse embryonic stem cells by substrate stiffness and topography. Biomaterials.

